# Tracking social provenance in chains of retweets

**DOI:** 10.1007/s10115-023-01878-7

**Published:** 2023-05-09

**Authors:** Sara Migliorini, Mauro Gambini, Elisa Quintarelli, Alberto Belussi

**Affiliations:** Department of Computer Science, University of Verona, Strada Le Grazie, 15, 37134 Verona, Italy

**Keywords:** Data provenance, Information discovery, Constraint propagation, Path consistency algorithm

## Abstract

In the era of massive sharing of information, the term social provenance is used to denote the ownership, source or origin of a piece of information which has been propagated through social media. Tracking the provenance of information is becoming increasingly important as social platforms acquire more relevance as source of news. In this scenario, Twitter is considered one of the most important social networks for information sharing and dissemination which can be accelerated through the use of retweets and quotes. However, the Twitter API does not provide a complete tracking of the retweet chains, since only the connection between a retweet and the original post is stored, while all the intermediate connections are lost. This can limit the ability to track the diffusion of information as well as the estimation of the importance of specific users, who can rapidly become influencers, in the news dissemination. This paper proposes an innovative approach for rebuilding the possible chains of retweets and also providing an estimation of the contributions given by each user in the information spread. For this purpose, we define the concept of Provenance Constraint Network and a modified version of the Path Consistency Algorithm. An application of the proposed technique to a real-world dataset is presented at the end of the paper.

## Introduction

The amount of information shared through social media is increasing in recent years, since they are becoming more popular than traditional media as source of news. Online newspapers, social networks, and content-sharing platforms are everyday flooded by multimedia content describing the world events of major interest. In this context Twitter is certainly one the mostly used source of information. It counts about 330 million monthly active users which span from individuals to official institutional accounts.

One of the main benefits introduced by this kind of media is that information can be shared and disseminated all over the network very easily. However, this introduces new problems related to the tracking of the information diffusion [1, 2] and the origin (provenance) of the information. For this reason, beside to the concept of data provenance [3–5], the term **social provenance** [6] has been introduced to inform about the ownership, source or origins of a given piece of information that propagates in a social network.

Twitter provides two important means for increasing the spread of information shared through a post (called tweet): retweets and quotes. The difference between the two is essentially the fact that the former simply reposts a previous tweet without any modification, while the latter adds some specific comments to the original content. For this reason, a retweet is considered an endorsement of the original post, while a quote can both provide support or express a different idea.

In this scenario it is crucial to track the diffusion of information [1, 2] as well as to estimate the importance of specific users in spreading the original message, for example with the aim of recommending friends and followers, or to infer users’ interests, needs, and political leaning [7]. However, the Twitter API does not provide a complete description of a retweet propagation path: the only information carried by a retweet is a link to the original tweet, whereas possible intermediate steps are lost, since no information are stored about them [8]. In other words, second-order retweets are treated as retweets of the original tweet and the retweet chains are not represented.

In this paper we propose a method for rebuilding the complete retweet chain by considering both temporal relations between posts and social connections between users. It uses a methodology inspired by the Temporal Constraint Network [9], for the construction of the interaction graph, and a modified version of the Path Consistency Algorithm [10], for the constraint propagation. More specifically, we introduce the concept of *Provenance Constraint Network* (*PCN*) where nodes represent tweets (or retweets) and edges are authorship constraints. An *authorship constraint* is used to track the complete possible provenance of a retweet. It not only specifies the set of users which likely contribute to the current post through a previous tweet or retweet, but also assigns to each of them a degree of ownership. In other words, all possible contributions are estimated and weighted based on the temporal relations between posts and the social relations between users. The basic idea is that if a user $u_1$ posts a retweet $RT_1$ of a tweet *T* created by *u*, but the social connection between $u_1$ and *u* is weaker than the connection with the author $u_2$ of another retweet $RT_2$ of *T* older than $RT_1$, then it is more likely that $RT_1$ originated from $RT_2$ rather than *T*.

Several techniques have been proposed in literature in order to generate retweet cascade graphs. However, all of them concentrate in the identification for each retweet of the most probable source connection, discarding all the other ones. Moreover, these approaches are not able to prevent the loss of important connections and this can be worsen by the absence of real benchmarks on which the proposed techniques can be validated and refined. Conversely, in this paper we propose a different approach, because we try to reconstruct all possible connections and weight them on the basis of temporal and social relations. In addition, the use of a constraint propagation approach allows us to propagate derived provenance information inside the network.

The overall contribution of the paper is manifold: (i) we introduce a formalism, called Provenance Constraint Network, for modeling the social and temporal connections among tweets, (ii) we propose a modified version of the propagation operations used by the Path Consistency Algorithm which deals with provenance information, (iii) we define a MapReduce implementation of the propagation algorithm which takes care of the semantical characteristics of a PCN in order to reduce the computational cost, and (iv) we introduce a set of metrics for evaluating the goodness of the obtained results in absence of a ground truth.

The remainder of the paper is organized as follows: Sect. 2 discusses some previous contributions about information propagation on Twitter, Sect. 3 provides a complete formalization of the addressed problem, Sect. 4 introduces the proposed solution from the construction of a PCN to the definition of a MapReduce version of the Path Consistency Algorithm, Sect. 5 reports some experiments performed on a real-world Twitter dataset about COVID posts, and finally Sect. 6 summarizes the obtained results and discusses some future extensions.

## Related work

The problem of social provenance or information diffusion has been investigated only at conceptual level in [11]: the authors propose PROVE-SAID, a unified conceptual model that provides concepts and definitions to deal with information diffusion and provenance in heterogeneous environments. In [12] the authors propose a social provenance framework for Twitter data, modeled in the NoSQL graph database Neo4j, but they do not provide an algorithm to translate data produced by Twitter API into their model.

In [13, 14] the authors propose mathematical models for predicting the retweet dynamics based on both temporal and social network information. In particular, in [13] the joint use of these two kinds of information is the one that produces the most reliable predictions about cascade dynamics, whereas in [14] the authors examine dynamics of tie strength, in terms of reciprocity, temporality, and context-awareness through social networks and propose a general model to predict the repliers and retweeters of a particular tweet considering friendship dynamics. For this reason, this paper proposes an approach based on both temporal and social information for deriving social provenance in retweet chains.

The method proposed in [8] for modeling retweet cascade graphs is based on the estimation of the interaction strength between each couple of users. Such metric is measured on the basis of the analysis of previous retweets, quotes and replies, as well as the consideration of the friend and follower sets. However, this method requires to retrieve for each group of retweets the most recent timeline of all involved users, and this can be a little cumbersome in extensive analysis. Other alternative techniques which consider the impact of the social relationships in the reconstruction of the retweet chains are described in [15, 16]. However, these methods measure the strength of social relationships only in terms of retweet dynamics, but this is not a complete measure, since no intermediate steps are registered in a chain of retweets.

At the best of our knowledge, given an original tweet and a set of its retweets, the main objective of the techniques proposed in literature is to identify for each retweet its actual source (the original tweet or another retweet) by using some heuristics based on the time and social interaction between the users. However, all these techniques are prone to errors and some important connections can be lost, also due to the absence of a base truth. Conversely, in this paper we define a technique which tries to estimate the likelihood of each possible connection and maintain a complete network of them.

The proposed technique is sufficiently generic to be enriched with other criteria or approaches for defining the interaction strengths between pairs of users, such as location information [17], or tweeting behavior [18]. In other words, any sophisticated formula for computing the interaction strengths can be easily plugged in the propagation algorithm.

A related but inverse problem, is the identification of the source of a rumor in an online social network. In this case, we have to track of the information spread and we want to identify the common source of information that originates the rumor [19–21]. Conversely, in our case, we do not want to identify the source of the information, since it is the only knowledge that we have (i.e., the original tweet) and we want to identify the possible chain of spread (i.e., the network of transmission) which is lost in Twitter. However, the adopted approaches share some similarities with the one proposed in this paper, which is the application of a probabilistic approach and the use of additional contextual information, like the network topology (i.e., social connections) and the speed of spreading (i.e., temporal aspects).

An approach similar to the one proposed in this paper can be found in [22], in this case the aim was to identify and track the provenance of data and the ownership of information in the archaeological context. In this scenario, we do not have a spread of an identical piece of information among different network nodes, but we have an evolution of the original data or the combination of several knowledge in order to obtain a new one. Moreover, some degrees of vagueness are added and depend on the kind of data to be managed, not on the kind of connections in the network. Anyway, the idea to track all possible connections and give them a certain level of confidence are common to both approaches.

## Problem formulation

This section provides a rigorous description of the considered problem. First of all, the concepts of tweet and user are formalized, then some observations are made about their possible connections, and finally the notion of authorship is introduced.

The term *tweet* is generically used to denote different kinds of posts, in particular: *general tweets*, *retweets* and the *quotes*.^1^ While a general tweet is an original content produced by a well recognized user, retweets and quotes are two ways to re-post the content of another tweet. The difference between a retweet and a quote is that the latter allows to add a comment to the mentioned tweet. For the purposes of this paper, we can safely neglect this last distinction, so anytime we use the term retweet, we can refer to either a retweet or a quote without distinction.

### Definition 1

*(Tweet)* A tweet *T* can be briefly represented as a tuple $\langle \textsf{id}, \textsf{user},$ $\textsf{timestamp}, \textsf{text}, \mathsf {retweet\_of} \rangle$, where $\textsf{id}$ is a unique identifier for the tweet *T*, $\textsf{user}$ is the identifier of the user who posted *T*, $\textsf{timestamp}$ is post timestamp, $\textsf{text}$ is the tweet content, and finally $\mathsf {retweet\_of}$ is the identifier of the source tweet, if *T* is a retweet of another tweet $T'$, or the empty value otherwise.

Notice that the last property is what allows us to distinguish a general tweet from a retweet: a tweet *T* is a retweet if and only if the property $T.\mathsf {retweet\_of}$ is not empty.

Throughout the paper, the set of all tweets will be denoted as $\mathcal {T}$, the set of all retweets as $\mathcal {R}$, with $\mathcal {R} \subset \mathcal {T}$. Given a tweet $T \in \mathcal {T}$, the set of retweets of *T* is denoted as $T.\mathcal {R}$.

### Definition 2

*(User)* A user *u* can be synthetically represented by a tuple $\langle \textsf{id}, \textsf{username}, \textsf{Followers}, \textsf{Friends}\rangle$, where $\textsf{Followers}$ is the set of users that are following *u*, while $\textsf{Friends}$ is the set of users that *u* is following.

The set of all users will be denoted as $\mathcal {U}$. Given a user $u \in \mathcal {U}$, $u.\textsf{Followers} \subseteq \mathcal {U}$ and $u.\textsf{Friends} \subseteq \mathcal {U}$. Moreover, given two users $u,w \in \mathcal {U}$, four different scenarios can be recognized: (i) $w \in u\cdot \textsf{Followers} \wedge w \notin u\cdot \textsf{Friends}$, *w* is following *u* but not vice versa, (ii) $w \in u\cdot \textsf{Friends} \wedge w \notin u\cdot \textsf{Followers}$, *u* is following *w* but not vice versa, (iii) $w \in u\cdot \textsf{Followers} \wedge w \in u\cdot \textsf{Friends}$, *u* and *w* are following each other, finally (iv) $w \notin u\cdot \textsf{Followers} \wedge w \notin u\cdot \textsf{Friends}$, *u* and *w* are not following each other at all.

**Fig. 1:**
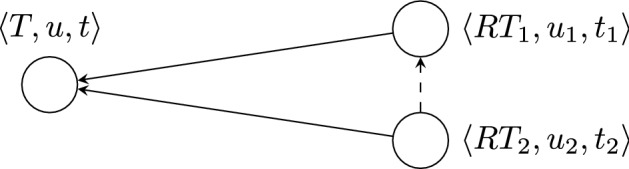
Example of connection between a general tweet *T* and two of its retweets $RT_1$ and $RT_2$. In the tuple $\langle T, u, t \rangle$, *T* is the tweet identifier, *u* is the user identifier and *t* is the tweet timestamp. The solid arrows represent connections stored in the Twitter API, while the dashed arrow is a derived one

Let us consider the situation depicted in Fig. 1; in this case we have a general tweet *T* with two retweets $RT_1$ and $RT_2$. Each tweet is represented by a node and an edge connects a retweet $RT_i$ to its original tweet *T*, so the edge represents the property $RT_i.\mathsf {retweet\_of}$. The label of the node $\langle T, u, t \rangle$ includes only the id, user and timestamp of the tweet, respectively. In accordance to what is stored by the Twitter platform, both $RT_1$ and $RT_2$ are connected only to the original general tweet *T* (see the two solid arrows from the retweets to the general tweet *T*). However, the following observations can be made.

### Observation 3.1

Given two retweets $RT_1$ and $RT_2$ of the same general tweet *T*, if the author $u_2$ of $RT_2$ is a follower of the user $u_1$, who is the author of $RT_1$, and the timestamp $t_2$ of $RT_2$ is greater than the timestamp $t_1$ of $RT_1$, then it is possible that $RT_2$ is a retweet of $RT_1$, rather than a retweet of the original general tweet *T*.

$$\begin{aligned}{} & {} \forall RT_1, RT_2 \in \mathcal {R} \,( \exists T\in \mathcal {T} \\{} & {} \quad (RT_1\in T\cdot \mathcal {R} \wedge RT_2\in T\cdot \mathcal {R} \,\wedge \\{} & {} \quad u_2 \in u_1.\textsf{followers} \,\wedge t_2 > t_1 ) \Rightarrow RT_2 \in RT_1.\widetilde{\mathcal {R}} ) \end{aligned}$$

where $RT.\widetilde{\mathcal {R}}$ is the estimated set of retweets of a retweet *RT*.

In Fig. 1, $RT_1.\widetilde{\mathcal {R}}$ is represented by the dashed edge connecting the node $RT_2$ with $RT_1$.

In the attempt to identify chains of retweets, previous proposals typically adopt strategies based on social network information (i.e., friends and followers) in conjunction with temporal information [23]; in particular they exploited the fact that users tend to interact more often with newer tweets [18], and thus, a user is more likely to retweet the last tweet of a friend [14].

### Observation 3.2

Given a retweet $RT_2$ performed by a user $u_2$ at timestamp $t_2$ and related to an original general tweet *T*, and a set *S* of users such that

$$\begin{aligned}{} & {} S = \{ s \in \mathcal {U} \mid u_2 \in s.\textsf{Followers} \;\wedge \\{} & {} \quad \exists RT_i \in T.\mathcal {R} \,(RT_i.\textsf{user} = s \,\wedge \\{} & {} \quad RT_i.\textsf{timestamp} < t_2 ) \} \end{aligned}$$

namely the set of users which are followed by $u_2$ and have posted a retweet of *T* prior to $u_2$. Then, the likelihood that $RT_2$ is a retweet of any $RT_i\in S$ depends on the cardinality of *S* and the distance between the timestamp of $RT_2$ and $RT_i$. The rationale is that if more than one followed users have performed a retweet of the same tweet, it is more likely that $u_2$ has taken the last of them as source of $RT_2$.

Another aspect to be considered is that the Twitter platform has recently changed its policies: it does not show content based on a simple reverse chronological order, but it also considers trend topics and their importance. More specifically, the likelihood that a retweet is a source of another retweet could depend on the connection (social interaction) between the two users.

### Observation 3.3

Given a user *u* and the set of her friends $F = u.\textsf{Friends}$, the interaction strength (IS) between *u* and $u_i \in F$ is directly proportional to the amount of mutual social activities, measured as the number of retweets, quotes and replies performed by one w.r.t. the tweets of the other one, or vice-versa:

1 $$\begin{aligned}&\forall u_i, u_j \in u.\textsf{Friends} \,( IS(u,u_i)> IS(u,u_j) \iff \nonumber \\&\quad |\textsf{connections}(u,u_i)| > |\textsf{connections}(u,u_j)| ) \end{aligned}$$

where $\textsf{connections}(u,u_i)$ is the set composed of all retweets, quotes and replies performed by *u* towards tweets from $u_i$, and vice-versa.

Given the above three observations, we can define the concept of *Provenance Constraint Network (PCN)*, which represents the first contribution of the paper.

### Definition 3

*(Provenance Constraint Network)* A *Provenance Constraint Network (PCN)* $\mathcal {N}$ is a tuple $\langle \mathcal {X}, \mathcal {C} \rangle$, where $\mathcal {X}$ is a set of nodes representing tweets, and $\mathcal {C}$ is a set of edges defining binary constraints between pair of nodes. In particular, each edge in $\mathcal {C}$ from a tweet node $T_i$ to a tweet node $T_j$ defines an authorship constraint for $T_i$ deriving from its relation with $T_j$. Each edge in $\mathcal {C}$ is represented as a tuple: $\langle T_i, T_j, C_{ij} \rangle$, where $C_{ij}$ is an authorship constraint as defined below.

### Definition 4

*(Authorship Constraint)* Given two tweet nodes $T_i$ and $T_j$, an *authorship constraint* $C_{ij}$ from $T_i$ to $T_j$ is represented as a set of tuples $\{(u_h,[s_h,e_h]),\dots \}$, called *authorship statements*, where $u_h \in \mathcal {U}$ is a user involved in the authorship of $T_i$ and $[s_h,e_h] \in \mathbb {R}^2$ is the corresponding degree of ownership. Each degree of ownership is represented as an interval of likelihood which extends from a minimum of $s_h$ to a maximum of $e_h$.

The following constraints are defined on the authorship statements composing an authorship constraint $C=\{(u_1,[s_1,e_1]),\dots ,$ $(u_n,[s_n,e_n])\}$:

2 $$\begin{aligned}{} & {} \forall C \in \mathcal {C}: \nonumber \\{} & {} \quad \forall i = 1, \dots n \,(s_i \ge 0) \,\wedge \end{aligned}$$

3 $$\begin{aligned}{} & {} \quad \forall i = 1, \dots n \,(s_i \le e_i) \,\wedge \end{aligned}$$

4 $$\begin{aligned}{} & {} \sum _{i=1}^{n} e_i = 1 \end{aligned}$$

In other words, the minimum likelihood of each authorship statement has to be greater than or equal to 0 (Eq. 2); the degree of ownership has to be a valid interval (Eq. 3), while the sum of the maximum likelihood has to be equal to 1 (Eq. 4).

## Proposed solution

This section illustrates the three algorithmic contributions of the paper: (i) the definition of a procedure for building a PCN given a set of general tweets and retweets (Sect. 4.1), (ii) the constraint propagation algorithm together with the customization of the operations on authorship constraints (Sect. 4.2), and finally (iii) a MapReduce implementation of the proposed propagation technique (Sect. 4.3).

In the next sections, we will refer to the following running example in order to better illustrate the PCN construction procedure and the constraint propagation operations.

### Example 1

Let us consider a set $\mathcal {T}$ containing two general tweets, $T_{1}$ and $T_{2}$, and five retweets, $RT_{1}$, $RT_{2}$, $RT_{3}$, $RT_{4}$ and $RT_{5}$. Their details are summarized in Table 1 where **ID** is the tweet identifier, **user** is the identifier of the user who posted the tweet, **timestamp** is the tweet timestamp, and **retweet-of** contains the identifier of the original tweet, if the current tweet is a retweet, or is left empty otherwise. In this case, $RT_{1}$, $RT_{2}$, $RT_{3}$ and $RT_{4}$ are retweets of $T_{1}$, while $RT_{5}$ is a retweet of $T_{2}$.

**Table 1:** Details of the tweets represented in Fig. 2

ID	User	Timestamp	Retweet-of
$T_{1}$	*u*	90	–
$RT_{1}$	$u_{1}$	110	$T_{1}$
$RT_{2}$	$u_{2}$	120	$T_{1}$
$RT_{3}$	$u_{3}$	100	$T_{1}$
$RT_{4}$	$u_{4}$	95	$T_{1}$
$T_{2}$	$u_{2}$	150	–
$RT_{5}$	$u_{3}$	170	$T_{2}$

### Construction of a PCN

Given a collection $\mathcal {T}$ including both general tweets and retweets regarding a considered period of time, a PCN $\mathcal {N}$ can be built by performing the operations summarized in Alg. 1. The definition of the following transformation rules is the second contribution of the paper and is an essential preparatory step for all the other operations. First of all, the set $\mathcal {X}$ of nodes for the network $\mathcal {N}$ is initialized (see line 4).

#### Definition 5

*(PCN Node)* Given a collection $\mathcal {T}$ of tweets, a node *n* is created and added to the network $\mathcal {N}$ for each $T\in \mathcal {T}$.

**Fig. 2:**
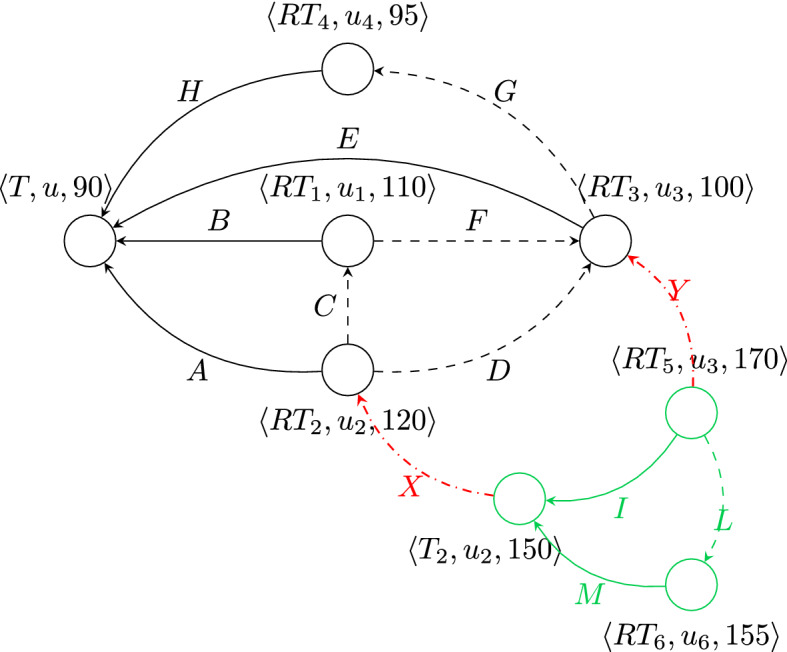
Example of PCN containing the tweets in Table 1

Given the situation introduced in Ex. 1, a node is created for each row of Table 1, as depicted in Fig. 2.

Once the set of nodes $\mathcal {X}$ of $\mathcal {N}$ has been built, we connect them by considering: (i) the link between a retweet and its original tweet, (ii) the temporal and social relations among the retweets of the same tweet, and (iii) the temporal connections among the tweets of the same authors.

Before proceeding with the edge definition, we introduce the notion of *Candidate Retweet Sources* (*CRS*) as the set of all tweets which can be considered a valid source for a current retweet *R*.

#### Definition 6

*(Candidate retweet sources)* Given a retweet $R \in \mathcal {R}$, the set of candidate retweet sources (CRS) $\mathcal {S}$ is defined as:

5 $$\begin{aligned}{} & {} \mathcal {S} = \{R.\mathsf {retweet\_of}\} \,\cup \nonumber \\{} & {} \quad \{S \in \mathcal {R} \mid S.\mathsf {retweet\_of} = R.\mathsf {retweet\_of} \,\wedge \nonumber \\{} & {} \quad S.\textsf{user} \in R.\textsf{Friends} \,\wedge \nonumber \\{} & {} \quad S.\textsf{timestamp} < R.\textsf{timestamp}\} \end{aligned}$$

In other words, besides to the original tweet $T = R.\mathsf {retweet\_of}$, the CRS contains all the other retweets of *T* which can be considered a source for *R* based on their social and temporal relations.

Given a retweet *R*, each connection between *R* and an element *S* in $\mathcal {S}$ can be weighted based on (i) the number of elements in $\mathcal {S}$ and (ii) the temporal distance between *R* and *S*.

#### Definition 7

*(Interaction weight)* Given a retweet *R* and its set of candidate retweet sources $\mathcal {S}$, the interaction weights between *R* and any element $S \in \mathcal {S}$ is computed in the following way: (i) for each $S \in \mathcal {S}$ the relative temporal distance between *R* and *S* is determined as:

6 $$\begin{aligned} u_{rs} = \dfrac{(R.\textsf{timestamp}-S.\textsf{timestamp})}{\sum _{U\in \mathcal {S}} (R.\textsf{timestamp}-U.\textsf{timestamp} )} \end{aligned}$$

then (ii) $u_{rs}$ is transformed in order to obtain a weight which is inversely proportional to the computed temporal distance and such that the sum of all the weights is equal to 1:

7 $$\begin{aligned} w_{rs} = \dfrac{1/u_{rs}}{\sum _{u \in S} 1/u_{ru}} \end{aligned}$$

The interaction weights have been normalized for using it as components of authorship statements, as we will see in the following definitions.

The first kind of edge to be built is the one connecting each retweet *RT* to its original tweet *T* (see Alg. 1 line 9).

#### Definition 8

*(R-T Edge)* Given a general tweet $T \in \mathcal {T} {\setminus } \mathcal {R}$ and the set $T.\mathcal {R}$ of its retweets, for each $R \in T.\mathcal {R}$ an edge is added from *R* to *T* and labeled with the authorship constraint:

8 $$\begin{aligned} C_{rt} = \left\{ \begin{array}{ll} \{(T\cdot \textsf{user},[w_{rt},1])\} &{} \text {if } |\mathcal {S}| > 1 \\ \{(T\cdot \textsf{user},[0,1])\} &{} \text {otherwise} \end{array} \right. \end{aligned}$$

where the value $w_{rt}$ is computed as in Eq. 7 of Def. 7.

As you can notice, in case more than one possible sources are available for *R*, the initial minimum likelihood is set equal to the interaction weight between *R* and *T*. Indeed, in line to what has been mentioned in Observation 3.1, the likelihood interval associated to each edge has to be inversely proportional to the temporal distance between the two nodes, so that the tweet, which is temporally closest to *R*, has a greater value for the minimum likelihood. Conversely, in case the only possible source for *R* is represented by the original tweet *T*, the initial minimum likelihood is set equal to 0 with the aim to promote the following constraint propagation process. Finally, the initial value for the maximum likelihood is set equal to 1 at the beginning, since no additional information is available and we need to satisfy Eq. 4.

Relatively to the network in Fig. 2, we have five edges of this kind. They are depicted with a solid line (i.e., *A*, *B*, *E*, *H* and *I*), and their constraint labels are reported in Table 2.

**Table 2:** Constraint labels for the edges in Fig. 2

Edge	Source	Target	Constraint
*A*	$RT_2$	*T*	$\{(u,[0.18,1])\}$
*B*	$RT_1$	*T*	$\{(u, [0.33,1])\}$
*C*	$RT_2$	$RT_1$	$\{(u_{1},[0.55,1])\}$
*D*	$RT_2$	$RT_3$	$\{(u_{3},[0.27,1])\}$
*E*	$RT_3$	*T*	$\{(u, [0.33,1])\}$
*F*	$RT_1$	$RT_3$	$\{(u_{3},[0.67,1])\}$
*G*	$RT_3$	$RT_4$	$\{(u_{4},[0.67,1])\}$
*H*	$RT_4$	*T*	$\{(u,[0,1])\}$
*I*	$RT_5$	$T_2$	$\{(u_2,[0.43,1])\}$
*L*	$RT_5$	$RT_6$	$\{(u_6,[0.57,1])\}$
*M*	$RT_6$	$T_2$	$\{(u_2,[1,1])\}$
*X*	$T_2$	$RT_2$	$\{(u_2,[1,1])\}$
*Y*	$RT_5$	$RT_3$	$\{(u_3,[1,1])\}$

The second kind of edge is the one connecting each retweet *R* to its CRS set, excluded the original tweet (see Alg. 1 lines 11-20). In particular, in Alg. 1 line 10, function $\textsf{CRS}(T,R.\textsf{user},R.\textsf{timestamp})$ returns the CRS set $\mathcal {S}$ for *R* as in Def. 6.

#### Definition 9

*(R-R Edge)* Given a tweet $T \in \mathcal {T} {\setminus } \mathcal {R}$ and the set $T.\mathcal {R}$ of its retweets, an edge is added from $R_{i} \in T.\mathcal {R}$ to $R_{j} \in T.\mathcal {R}$ if and only if $R_{j} \in R_{i}.\textsf{Friends}$ and $R_{j}.\textsf{timestamp} < R_{i}.\textsf{timestamp}$. The label associated to this edge will contain the authorship constraint:

9 $$\begin{aligned} C_{r_{i}r_{j}} = \{(R_{j}.\textsf{user},[w_{r_{i}r_{j}},1])\} \end{aligned}$$

where the value $w_{r_i r_j}$ is computed as in Eq. 7 of Def. 7.


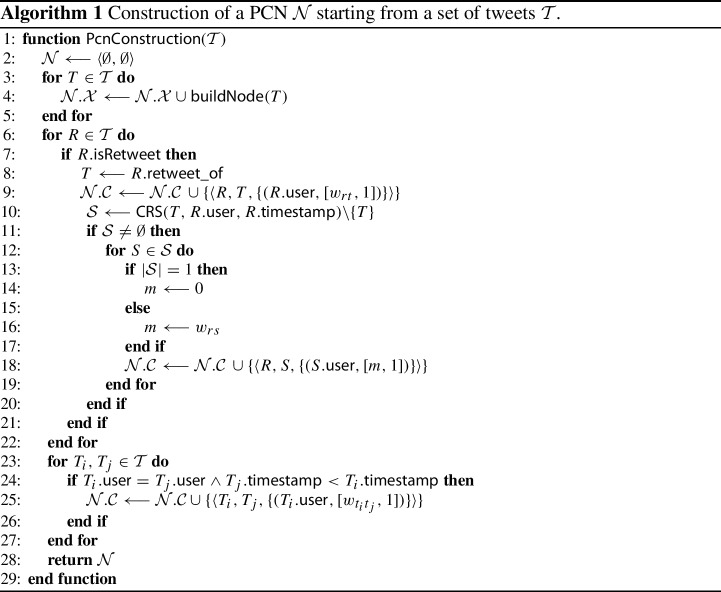


The same considerations made in Def. 8 relatively to the authorship constraint are also valid here. This kind of edges are depicted with dashed lines in Fig. 2 (i.e., *C*, *D*, *F*, *G*, *L*) and the constraint values are reported in Table 2.

The set of edges introduced until now has the effect to build a set of independent sub-networks, one for each original tweet *T*. The last kind of edge is the one which provides a link between different sub-networks, since it connects tweets performed by the same author and could be useful in order to strengthen the social relationships between users (see Alg. 1 lines 23-27).

#### Definition 10

*(T-T Edge)* Given two tweets $T_{i}, T_{j} \in \mathcal {T}$, an edge is added from $T_{i}$ to $T_{j}$ if and only if $T_{i}.\textsf{user} = T_{j}.\textsf{user}$ and $T_{j}.\textsf{timestamp}$ $< T_{i}.\textsf{timestamp}$. The edge will be labeled with the following constraint:

10 $$\begin{aligned} C_{t_{i}t_{j}} = \{(T_{j}.\textsf{user}, [1,1] )\} \end{aligned}$$

This last kind of edge is depicted in Fig. 2 as red dot-dashed lines (i.e., *X*, *Y*), while the associated constraints are reported in Table 2. As you can notice, the resulting PCN is composed of different sub-networks, each one originating from a different source tweet, which are connected with each other through *T-T* edges.

Given a PCN, some constraint propagation operations can be performed in order to reduce the uncertainty associated to the degree of ownership of each user appearing in a constraint. More specifically, the Path Consistency Algorithm [10] can be applied; it relies on the conjunction and compositions operations, which are redefined on authorship constraints in the following section.

### Constraint propagation

The Path Consistency Algorithm [10] is a classical technique used for constraint propagation with the main aim to reduce the variable domains after the arc-consistency is performed. In a PCN, such algorithm can be used to propagate the authorship and restrict the interval of likelihood associated to each user inside a constraint, namely to provide a more precise measure of their degree of ownership. The idea behind the Path Consistency Algorithm is very simple, given three nodes $x_i$, $x_j$ and $x_k$, such that there exists a constraint $C_{ij}$ between $x_i$ and $x_j$, a constraint $C_{ik}$ between $x_i$ and $x_k$, and a constraint $C_{kj}$ between $x_k$ and $x_j$ that completes the triangle, a new constraint can be derived between $x_i$ and $x_j$ by properly combining them.

#### Definition 11

*(Path Consistency Algorithm)* Given three nodes $x_i$, $x_k$ and $x_j$ of a PCN $\mathcal {N}$, a new constraint between $x_i$ and $x_j$ can be derived from the existing constraints by the path consistency algorithm as follows:

11 $$\begin{aligned} C_{ij}' = C_{ij} \otimes (C_{ik} \circ C_{kj}) \end{aligned}$$

where $C_{ij}$ is the constraint existing between $x_{i}$ and $x_{j}$, $C_{ik} \circ C_{kj}$ is the composition of two constraints, and $C_{ij} \otimes C$ is the conjunction.

In order to actually apply the path consistency algorithm to a PCN $\mathcal {N}$, it is necessary to define the operations of inversion, conjunction and composition between authorship constraints. The definition of these operations is the third contribution of the paper: they are essential for applying the constraint propagation and the derivation of new provenance knowledge.

#### Definition 12

*(inversion)* Given an authorship constraint $C_{ij} = \{(u_1,[s_1,e_1]),$ $\dots (u_n,[s_n,e_n])\}$ between two nodes $x_{i}$ and $x_{j}$ of a PCN, the constraint $C_{ij}^{-1}$ represents the equivalent constraint holding between $x_{j}$ and $x_i$. Such constraint can be obtained by making the inversion of intervals in each authorship statement:

12 $$\begin{aligned} C_{ij}^{-1} = \{(u_1,[-e_1,-s_1]),\dots ,(u_n,[-e_n,-s_n])\} \end{aligned}$$

The inversion operation could be particularly useful during the constraint propagation task, since in order to identify a possible reduction triangle, the inverse of a constraint can be required, as illustrated in the following example.

#### Example 2

Let us consider the network in Fig. 2, an update constraint *C* can be obtained as $C = C \otimes (D \circ F^{-1})$, in this case the inverse of the constraint *F* is necessary and $F^{-1} = \{(u_3,[-1,-0.67])\}$. The inverse of a single constraint may seem not to be particularly meaningful, but its importance appears when the complete propagation is performed.

#### Definition 13

*(composition* $\circ$*)* Given two authorship constraints $C_{ik} = \{(x_{1},[a_{1},b_{1}]),$ $\dots ,(x_{n},[a_{n},b_{n}])\}$ and $C_{kj} = \{(y_1,[c_1,d_1]),$ $\dots ,(y_m,[c_m,d_m])\}$, their composition $C_{ik} \circ C_{kj}$ is defined as follows:

13 $$\begin{aligned}&C_{ik} \circ C_{kj} = \{(u,[s,e]) \mid \nonumber \\&\quad \exists c \in C_{ik}(c.\textsf{user} = u \wedge \exists d \in C_{kj}(d.\textsf{user} = c.\textsf{user} \,\wedge \nonumber \\&\quad (s = c. s + d. s ) \wedge (e = c. e + d. e ))) \,\vee \nonumber \\&\quad \exists c \in C_{ik}(c.\textsf{user} = u \wedge \not \exists d \in C_{kj} (d.\textsf{user} = c.\textsf{user} \, \wedge \nonumber \\&\quad (s = c. s \wedge e = c. e))) \,\vee \nonumber \\&\quad \exists d \in C_{kj}(d.\textsf{user} = u \wedge \not \exists c \in C_{ik} (c.\textsf{user} = d.\textsf{user} \, \wedge \nonumber \\&\quad (s = d. s \wedge e = d. e)))\} \end{aligned}$$

In other words for each user appearing in both the original constraints, the degree of ownership in the composed constraint is the sum of the two degrees of ownership; otherwise, for each user appearing in only one of the two constraints, denoted as *c* (or *d*), the degree of ownership in *c* (or *d*) becomes the degree of the composed constraint.

#### Example 3

Let us consider again the situation in Fig. 2 and the propagation of the constraint *C* in Ex. 2. The composition of the constraints *D* and $F^{-1}$ produces the following constraint:

$$\begin{aligned} D \circ F^{-1}&= \{(u_{3},[0.27,1])\} \circ \{(u_3,[-1,-0.67])\} \\&= \{(u_{3},[-0.73,0.33])\} \end{aligned}$$

In this case, the two authorship statements regard the same author and their degrees of ownership are summed.

#### Definition 14

*(conjunction* $\otimes$*)* Given two authorship constraints $C_{ik} = \{(x_{1},[a_{1},b_{1}]),\dots ,(x_{n},[a_{n},b_{n}])\}$ and $C_{kj} = \{(y_1,[c_1,d_1]),$ $\dots ,(y_m,[c_m,d_m])\}$, their conjunction $C_{ik} \otimes C_{kj}$ is defined as follows ($u_i$ is the user associated to the source node $x_i$, involved in the constraint $C_{ik}$):

14 $$\begin{aligned}&C_{ik} \otimes C_{kj} = \{(u,[s,e]) \mid u \in u_i.\textsf{friends}. \, \wedge \nonumber \\&\quad (\exists c \in C_{ik} (c.\textsf{user} = u \, \wedge \exists d \in C_{kj} (d.\textsf{user} = c.\textsf{user} \, \wedge \nonumber \\&\quad s = \max \{0,\max \{c. s, d. s\}\} \wedge e = \max \{0,\min \{c. e, d. e\}\} )) \;\vee \nonumber \\&\quad \exists c \in C_{ik}(c.\textsf{user} = u \, \wedge \not \exists d \in C_{kj}(d.\textsf{user} = c.\textsf{user} \, \wedge \nonumber \\&\quad s = \max \{0,c. s\} \wedge e = \max \{0,c. e\})) \;\vee \nonumber \\&\quad \exists d \in C_{kj}(d. \textsf{user} = u \, \wedge \not \exists c \in C_{ik}(c. \textsf{user} = d. \textsf{user} \, \wedge \nonumber \\&\quad s = \max \{0,d. s\} \wedge e = \max \{0,d. e\})))\} \end{aligned}$$

In other words, the conjunction operation computes, for each user *u* belonging to the set of friends of the source user $u_i$, the corresponding degree of ownership as follows: if *u* appears in both the considered constraints, their degrees of ownership are summed; otherwise, if *u* appears only in one of the two constraints, its original degree of ownership is taken.

#### Example 4

Let us consider again the situation in Fig. 2 and the propagation of the constraint *C* in Ex. 3. The conjunction of *C* with the result of $(D \circ F^{-1})$ produces the following result:

$$\begin{aligned} C \otimes (D \circ F^{-1})&= \{(u_{1}, [0.55,1])\} \otimes \{(u_{3},[-0.73,0.33])\} \\&= \{(u_{1}, [0.55,1]), (u_3,[0,0.33]) \} \end{aligned}$$

Since both $u_1$ and $u_3$ belong to the set of friends of $u_2$, the author of the source tweet for constraint *C*, the conjunction maintains both of them, and for $u_3$ the left extreme of the interval becomes 0.

#### Example 5

Let us consider again the situation in Fig. 2 and the propagation of the constraint *E* as $E=E \otimes (G \circ H)$:

$$\begin{aligned} E&= \{(u,[0.33,1]\} \otimes (\{(u_4,[0.67,1])\} \circ \{(u,[0,1])\}) \\&= \{(u,[0.33,1]\} \otimes \{(u_4,[0.67,1]),(u,[0,1])\} \\&= \{(u,[0.33,1],(u_4,[0.67,1])\} \end{aligned}$$

In this case the user *u* appears in both constraints, so the intersection between their degrees of ownership is taken in the result.

#### Example 6

Let us consider again the situation in Fig. 2 and the propagation of the constraint *B* as $B=B \otimes (F \circ E)$ after the update of *E* as in Ex. 5:

$$\begin{aligned}&B = \{(u,[0.33,1])\} \otimes (\{(u_3,[0.67,1])\} \circ \{(u,[0.33,1],(u_4,[0.67,1])\}) \\&\quad = \{(u,[0.33,1]),(u_3,[0.67,1]),(u_4,[0.67,1])\} \\&\quad = \{(u,[0.33,1]),(u_3,[0.67,1])\} \end{aligned}$$

In this case the authorship statement related to $u_4$ has been removed, since $u_4$ is not a friend of $u_1$.

#### Example 7

Let us consider again the situation in Fig. 2 and the propagation of the constraint *A* as $A = A \otimes (C \circ B)$, after update of constraint B in Ex. 6:

$$\begin{aligned} A&= \{(u,[0.18,1])\} \otimes \\&\qquad ( \{(u_{1}, [0.55,1]), (u_3,[0,0.33]) \} \;\circ \\&\qquad \{(u,[0.33,1],(u_3,[0.67,1])\}) \\&= \{(u,[0.18,1])\} \otimes \{(u,[0.33,1]),(u_1,[0.55,1]),(u_3,[0.67,1.33])\} \\&= \{(u,[0.33,1]),(u_1,[0.55,1]),(u_3,[0.67,1.33])\} \end{aligned}$$

The previous examples bring out the fact that the resulting constraint could not satisfy Eq. 4. Therefore, the following normalization operation is needed in order to complete the propagation and produce the final result.

#### Definition 15

*(normalization)* Given an authorship constraint $C = \{(u_1,[s_1,e_1]),$ $\dots (u_n,[s_n,e_n])\}$ between two nodes *x* and *y* of a PCN, the normalization *normalize*(*C*) is the operation that properly modifies the degree of ownership associated to each author in such a way that the constraint in Eq. 4 is satisfied:

$$\begin{aligned} normalize(C)&= \left\{ c' \mid \exists c \in C \,(c'. user = c. user \, \wedge \right. \\ c'. s&= \left. \left. \dfrac{c. s}{\sum _{d \in C} d. e} \,\wedge c'. e = \dfrac{c. e}{\sum _{d \in C} d. e} \right) \right\} \end{aligned}$$

#### Example 8

Let us consider the result of the propagation obtained in Ex. 7, the final value for constraint *A*, after normalization, becomes:

15 $$\begin{aligned} A = \{(u,[0.09,0.30]),(u_1,[0.16,0.30]),(u_3,[0.20,0.40])\} \end{aligned}$$

As you can notice from the PCN in Fig. 2, we can identify triangles for the application of the Path Consistency Algorithm only inside the same sub-network. Conversely, *T-T* edges (like *X* and *Y*) which connect two distinct sub-networks are never part of an interesting triangle. Indeed, possible triangles could involve: (i) nodes contained inside the same sub-network, or (ii) nodes contained in different sub-networks and connected through only *T-T* edges. In the last case, the nodes are related to the same user *u* and the constraints have all the form (*u*, [1, 1]), so no actual propagation can be performed. However, *T-T* edges can be useful for identifying the strength of the social connection between authors. Let us consider the situation in Fig. 2, we can observe that edges *X* and *Y* connect two sub-networks by linking two nodes related to $u_2$ and two nodes of $u_3$, respectively. Moreover, $u_2$ and $u_3$ are connected in both sub-networks, creating together a loop. This situation identifies a stronger connection between $u_2$ and $u_3$, because there is more than one case in which they are socially connected.

In order to exploit such kind of connections, we need to formalize the notion of PCN sub-network and PCN loop.

#### Definition 16

*(PCN sub-network)* Given a PCN $\mathcal {N}=\langle \mathcal {X},\mathcal {C}\rangle$, a sub-network $\mathcal {M} = \langle \mathcal {Y}, \mathcal {D} \rangle$ of $\mathcal {N}$ can be defined as follows:

16 $$\begin{aligned}&\mathcal {Y} \subseteq \mathcal {X} \end{aligned}$$

17 $$\begin{aligned}&\mathcal {D} \subseteq \mathcal {C} \wedge \forall d \in \mathcal {D} ( d \text { is of kind } \textit{R-T} \text { or } \textit{R-R} ) \end{aligned}$$

With reference to the PCN depicted in Fig. 2, two sub-networks can be identified: $\mathcal {M}_1 = \langle \{T, RT_1, RT_2, RT_3, RT_4\}, \{A,B,C,D,E,F,$ $G,H\}\rangle$ and $\mathcal {M}_2 = \langle \{T_2,RT_5,RT_6\}, \{I,L,M\} \rangle$. These two sub-networks are connected through two *T-T* edges forming a loop composed of the nodes $RT_2$, $RT_3$, $T_2$ and $RT_5$.

#### Definition 17

*(PCN loop)* Given a PCN $\mathcal {N}$ in which we can identify two sub-networks $\mathcal {M}_i$ and $\mathcal {M}_j$, a *loop* is a tuple

18 $$\begin{aligned} \langle x_h, x_k, x_m, x_n \rangle \in \mathcal {X}^4 \end{aligned}$$

such that:

19 $$\begin{aligned}&x_h, x_k \in \mathcal {M}_i \wedge x_m, x_n \in \mathcal {M}_j \, \wedge \nonumber \\&\quad x_h. \textsf{user} \ne x_{k}. \textsf{user} \,\wedge \nonumber \\&\quad x_h. \textsf{user} = x_{m}. \textsf{user} \,\wedge x_k. \textsf{user} = x_{n}. \textsf{user} \,\wedge \nonumber \\&\quad ( (x_h,x_k) \in \mathcal {M}_i. \mathcal {C} \,\vee (x_k,x_h) \in \mathcal {M}_i. \mathcal {C} ) \,\wedge \nonumber \\&\quad ( (x_m,x_n) \in \mathcal {M}_j. \mathcal {C} \,\vee (x_n,x_m) \in \mathcal {M}_j. \mathcal {C} ) \,\wedge \nonumber \\&\quad (x_h,x_m) \in \mathcal {N}. \mathcal {C} \,\wedge (x_k,x_n) \in \mathcal {N}. \mathcal {C} \end{aligned}$$

In Fig. 2 nodes $\langle RT_2, RT_3, RT_5, T_2\rangle$ form a loop, indeed (i) $RT_2, RT_3 \in \mathcal {M}_1$ are connected through an *R-R* edge in $\mathcal {M}_1$, (ii) $RT_5, T_2 \in \mathcal {M}_2$ are connected through an *R-T* edge in $\mathcal {M}_2$, (iii) while $T_2$ and $RT_2$ have the same author (i.e., $u_2$) and are connected through a *T-T* edge, and finally, $RT_5$ and $RT_3$ have the same author (i.e., $u_3$) and are connected through a *T-T* edge.

We can use the presence of a loop for increasing the degree of ownership of the referenced user inside the constraints of each sub-network, as described below.

#### Definition 18

*(enhancement)* Given a network $\mathcal {N}$ and two users $u_i, u_j \in \mathcal {U}$ such that $\mathcal {L}_{ij}$ is the set of loops involving $u_i$ and $u_j$:

20 $$\begin{aligned}&\mathcal {L}_{ij} = \{\langle x_h, x_k,x_m,x_n\rangle \mid \nonumber \\&\quad x_h, x_k \in \mathcal {M}_{i} \wedge x_m, x_n \in \mathcal {M}_{j} \wedge \nonumber \\&\quad x_h. \textsf{user} = x_m. \textsf{user} = u_i \wedge \nonumber \\&\quad x_k. \textsf{user} = x_n. \textsf{user} = u_j \} \end{aligned}$$

where $\mathcal {M}_{i}$ and $\mathcal {M}_{j}$ are two sub-networks of $\mathcal {N}$. For each constraint $C \in \mathcal {N}\cdot \mathcal {C}$ from node $x_i$ to node $x_j$ such that the author of $x_i$ is $u_i$ and the author of $x_j$ is $u_j$ (or vice versa), the degree of ownership associated to $u_j$ in *C* can be enhanced by a weight $e_{ij}$ computed as:

21 $$\begin{aligned} e_{ij} = 1 +\dfrac{|\mathcal {L}_{ij}|}{|\mathcal {N}\cdot \mathcal {C}_{i}|} \end{aligned}$$

where $\mathcal {N}\cdot \mathcal {C}_{i}$ is the set of edges in $\mathcal {N}$ which involve the node $x_i$.

#### Example 9

Let us consider the situation in Fig. 2 and the updated constraint *A* computed in Ex. 7, in this case the statement regarding $$u_3$$ can be enhanced due to the presence of a loop involving $$u_2$$ (the source user) and $$u_3$$. The weight is equal to $$e_{23} = 1 + 1/4 = $$ 1.25 and the updated constraint (after the enhancement and normalization) becomes:

$$\begin{aligned}&A = \{(u,[0.09,0.30]),(u_1,[0.16,0.30]),(u_3,[0.25,0.50])\} \\&\quad = \{(u,[0.08,0.27],(u_1,[0.15,0.27]),(u_3,[0.23,0.46])\} \end{aligned}$$

Given the above definitions, a constraint propagation algorithm can be applied inside and outside the sub-networks of $\mathcal {N}$. Once the propagation has been done, the resulting network contains in each *R-T* edge an authorship constraint describing all the possible source of authorship for the source node. More specifically, with reference to the situation in Ex. 9, after the application of the constraint propagation algorithm, the edge *A* will finally contain the following constraint:

$$\begin{aligned} A = \{(u,[0.16,0.24],(u_1,[0.20,0.29]),(u_3,[0.28,0.47])\} \end{aligned}$$

which means that as regards to the retweet $RT_2$ posted by $u_2$, the users involved in its social provenance are: *u*, $u_1$ and $u_3$. The greater degree of ownership is associated to $u_3$, even if the temporal distance between $RT_2$ and $RT_1$ is less than the temporal distance between $RT_2$ and $RT_3$. This is also due to their social interaction provided by the presence of the PCN loop. Conversely, $u_1$ has a degree of ownership greater than to *u*, due to the smaller temporal distance between the two retweets.

Given all these considerations, the following section presents a MapReduce implementation of the constraint propagation procedure which includes both the computation of the enhancements and the parallel application of the Path Consistency Algorithm.

### MapReduce constraint propagation

The classical Path Consistency Algorithm is characterized by a high computational complexity which makes its application unpractical in many real-world problems. Indeed, its theoretical complexity is equal to $O(n^3k^5)$, where *n* is the number of nodes and *k* is the number of edges. However, as we have already observed in the previous section, a PCN is typically composed of several highly-connected sub-networks which can be linked together by *T-T* edges which do not directly participate to the constraint propagation. This particular structure of a PCN suggests that the constraint propagation can be actually performed by considering each sub-network alone in a parallel way.
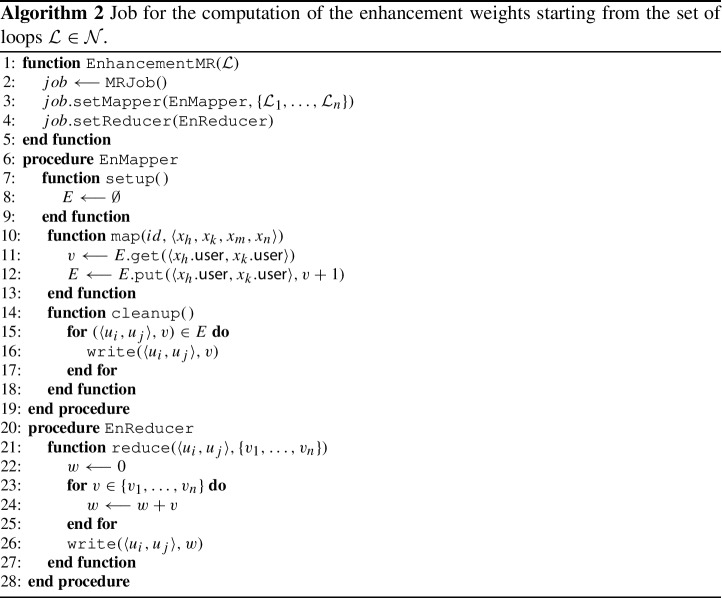


Therefore, we assume that the overall network $\mathcal {N}$ produced by Alg. 1 has been stored as two separated outputs: (i) one containing the subnetworks that will become the input of the path consistency job in Alg. 3 as we will discuss later, and (ii) one containing the loops $\mathcal {L}$ which connect the distinct subnetworks. This last output is useful for computing the social interactions between pairs of users, and we use them inside the MapReduce job in Alg. 2.
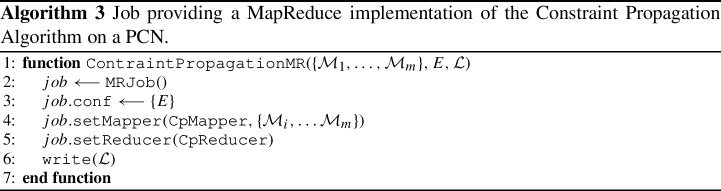


In more details, in the job of Alg. 2, each mapper receives and processes a subset $\mathcal {L}_i$ of the network loops $\mathcal {L}$ and essentially counts the user interactions contained in it (lines 6-18). This counter is stored into an auxiliary data structure *E*, which is initialized in the setup method. Each $\mathcal {L}_i$ represents a split in the MapReduce terminology (see Alg. 2 line 3). Each mapper has a complexity equal to $O(|\mathcal {L}_i|)$, namely equal to the number of loops contained inside the same split $\mathcal {L}_i$. 
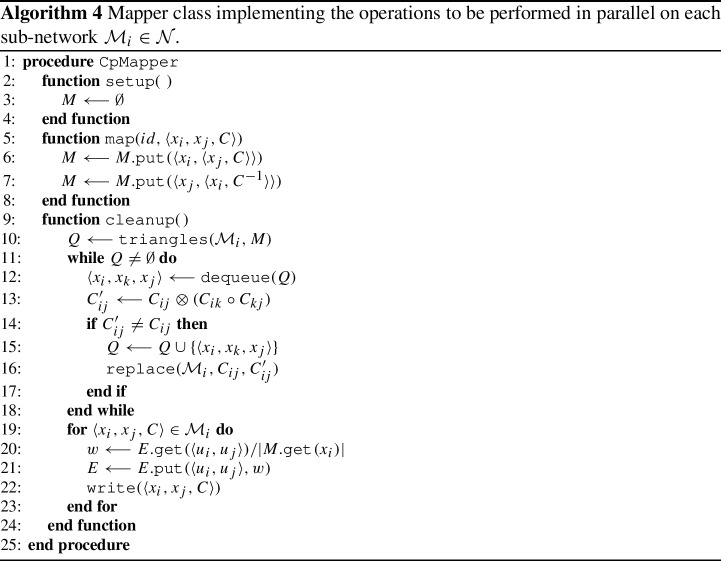

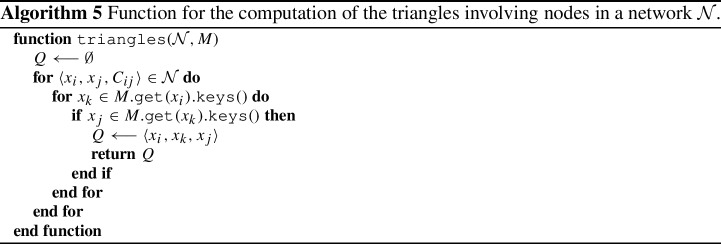


The reducer (line 20–27) simply combines the partial results by summing-up the partial counters produced by the mappers for each pair of users. The final output is an associative array which returns for each pair of users a value representing the number of loops involving them. The reducer has a complexity equal to $O(|\mathcal {U}|\times |\mathcal {U}|)$, namely equal to the number of possible pairs of users, since it has to process each of them for computing the final result.

Given the computation of the enhancement weights, we can proceed with the definition of the MapReduce job for the constraint propagation. The overall job is reported in Alg. 3 and assumes that the network $\mathcal {N}$ has been subdivided into a set of splits $\{\mathcal {M}_1,\dots \mathcal {M}_m\}$, each one representing a distinct subnetwork, by using an appropriate partitioning technique [24, 25]. The job is also aware of the associative array *E* which has been computed by the previous job and has been stored as a configuration parameter for latter uses inside the reduce phase. Finally, the set of loops $\mathcal {L}$ is given in order to finally reconstruct the overall network.

The implementations of the map and reduce phases of the job are illustrated in Alg. 4 and 6, respectively. The mappers assume that a sub-network is represented as a set of records with the form $\langle x_i, x_j, C_{ij}\rangle$ where $x_i$ is the source node, $x_j$ is the target node and $C_{ij} \in \mathcal {N}.\mathcal {C}$ is the constraint between $x_i$ and $x_j$. Alg. 4 illustrates the operations performed by the mappers on each sub-network $\mathcal {M}_i \in \mathcal {N}$. During a preliminary setup, an auxiliary data structure *M* is initialized: it will be populated inside the map method by storing each edge in $\mathcal {M}_i$ as well as its inverse in an associative way. This data structure is particularly useful for the application of the path consistency algorithm, which is actually performed inside the cleanup. An auxiliary function, called $\textsf{triangles}$, is used to identify the possible triples of nodes on which the constraint propagation can be performed. Notice that at the end of the map phase, the computation of the enhancement weights is completed by dividing the value stored in *E* during the previous task by the number of edges involving the source node as in Eq. 21. The complexity of each mapper is dominated by the application of the classical Path Consistency Algorithm during the cleanup phase. Even if its complexity remains $O(n_i^3k_i^5)$, we can observe that in real-world applications $n_i \ll n$ and $k_i \ll k$, namely the number of nodes and edges contained in each sub-network is typically much less than the number of nodes and edges in the overall network. This has also some consequences on the scalability of the approach: an eventual increment in the network size is usually due to an increment of the number of sub-networks rather than an increment of their respective sizes.
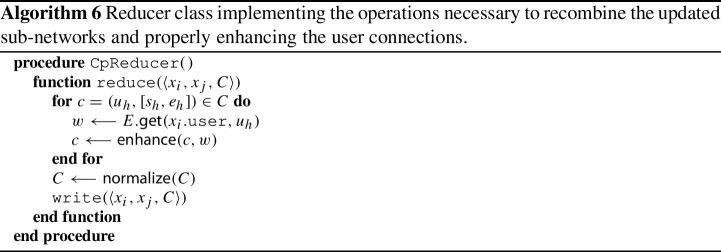


The reducer is illustrated in Alg. 6; it is responsible for rebuilding the overall network starting from the partial results produced by the maps. More specifically, it writes back the updated edges contained inside each subnetwork (i.e., the output produced by the mappers), after having properly enhanced them through the weights contained in the map *E* (see Alg. 6 line 5). After the enhancement of each authorship statement, a normalization of the overall constraint is performed in order to guarantee the satisfaction of Eq. 4. Notice that more than one reducer can be executed in parallel in order to produce the final result. The complexity of each reducer is $O(\sum |\mathcal {M}_i. \mathcal {C}|/r)$, where *r* is the total number of instantiated reducers: each reducer receives a portion of the edges computed by all the mappers (i.e., a portion of the edges contained in the sub-networks).

The last operation to be performed for completing the network reconstruction is the output of the *T-T* edges which connect different subnetworks and are not processed by the mappers. This is performed at the end of the main job (see Alg. 3 line 6) after the reducers completion.

## Case study

For the experimental phase, we consider a portion of dataset provided in [26] which contains a large collection of COVID-19 related tweets collected starting from January 2020. Since this dataset contains only synthetic data, it has been properly enriched (hydrated) to retrieve the necessary information, such as the indication of whether a post is a retweet or not, its timestamp and the reference to the original tweet, the user id and the list of her followers.

Given the mentioned set of tweets, we have extrapolated five different datasets with increasing sizes, in order to test the performances of the proposed approach. Table 3 reports the overall characteristics of these datasets such as the spanning time, the number of rows and the number of users.

**Table 3:** General metadata about the considered datasets

DS	Timespan	# Rows	# Users
$D_1$	2 days	565,478	246,374
$D_2$	3 days	747,197	375,247
$D_3$	4 days	864,759	492,130
$D_4$	8 days	1,226,996	668,488
$D_5$	10 days	1,369,578	739,350

Given the five datasets reported in Table 3, the procedure illustrated in Alg. 1 has been applied in order to obtain the corresponding PCNs. Table 4 reports some characteristics of the obtained networks, in particular: the number of nodes, the number of edges and the number of contained subnetworks. Notice that in the identification of the sub-networks, those with less than three nodes have been discarded, since they cannot contain any triangle useful for the propagation.

**Table 4:** Metadata about the obtained PCNs

DS	# Nodes		# Edges			# Subnetworks
	Tweets	Retweets	RT	RR	TT	
$D_1$	6,033	32,038	32,038	240	82,959	32
$D_2$	9,272	54,890	54,890	408	165,807	48
$D_3$	11,345	70,373	70,373	563	224,880	62
$D_4$	18,048	113,304	113,304	1,122	498,284	120
$D_5$	21,883	132,464	132,464	1,547	770,066	148

Finally, Table 5 reports some summary information about the subnetworks and the HDFS splits containing them. As discussed in [24], in order to better exploit the advantages induced by a MapReduce approach, it is necessary that the applied partitioning technique creates balanced splits, which means having a uniform amount of work to be done in parallel by each mapper. In our case, this can happen only if the identified sub-networks have substantially a similar size.

**Table 5:** Statistics about the sub-network splits regarding the considered datasets

DS	Min edges	Max edges	Avg. edges	Avg. %RSD
$D_1$	5	2,169	226.16	173%
$D_2$	5	4,571	383.46	209%
$D_3$	5	5,322	398.39	151%
$D_4$	5	5,455	320.23	203%
$D_5$	5	5,467	282.80	196%

For this reason Table 5 reports some statistics about the obtained splits, such as the minimum, maximum and average number of edges (rows in the splits) as well as the relative standard deviation between the split sizes. From the obtained results, we can observe that at first glance the subnetworks are not well balanced. There are some very small subnetworks (i.e., with very few edges) and others that are very big. However, the average size and the average relative standard deviation indicate us that on average the size of the various subnetworks (i.e., the split sizes) does not differ more than 4 times. In other words, the number of sub-networks containing the minimum or the maximum amount of edges (outliers) is very small, while the others have a comparable size (i.e., the same order of magnitude), even if not exactly the same.

### Evaluation metrics

Evaluating the correctness of the derived information about social provenance is not a trivial task: as already highlighted in previous works, the absence of ground truth information prevents the use of standard evaluation metrics. In this paper we use an approach similar to the one applied in [8, 27], which consists in the definition of a set of metrics. However, in this case since we try to identify and evaluate all the possible provenances of a given post, the defined metrics cannot be directly applied and we have to slightly modify them. The definition of the following evaluation metrics is the last contribution of the paper. They represent an important tool for evaluating the performances of the approach in absence of a base truth.

#### Definition 19

*(Average constraint size)* Given a PCN $\mathcal {N} = \langle \mathcal {X}, \mathcal {C} \rangle$ the *Average Constraint Size* (*ACS*) measures the average number of authorship statements contained in the authorship constraints $\mathcal {C}$.

22 $$\begin{aligned} \textit{ACS}(\langle \mathcal {X}, \mathcal {C} \rangle ) = \dfrac{\sum _{K \in \mathcal {C}}|K|}{|\mathcal {C}|} \end{aligned}$$

where for each constraint $C \in \mathcal {C}$ the value |*C*| is the number of authorship statements in *C*.

In the proposed approach, the network obtained after the constraint propagation has an ACS higher than the one of the original network. Indeed, at the beginning, the network will contain only one authorship statement for each constraint. Conversely, after the application of the constraint propagation, more statements should be added in each constraint, meaning that additional information is now available.

#### Definition 20

*(Sparse node incidence)* Given a PCN $\mathcal {N} = \langle \mathcal {X}, \mathcal {C} \rangle$ the *Sparse Node Incidence* (*SPI*) is the number of retweets whose constraint size (i.e., number of statements in the constraint) is equal to one, divided by the overall number of retweets:

23 $$\begin{aligned} \textit{SPI}(\langle \mathcal {X}, \mathcal {C} \rangle ) = \dfrac{ |\{x \in \mathcal {X} \mid x. \textsf{type} = \textsf{RT} \,\wedge \, |C_{x,x. \mathsf {retweet\_of}}| = 1 \}| }{ |\{x \in \mathcal {X} \mid x. \textsf{type} = \textsf{RT}\}| } \end{aligned}$$

where $x\cdot \textsf{type}$ returns the type associated to a node $x \in \mathcal {X}$, namely $\textsf{RT}$ in case of a retweet or $\textsf{T}$ in case of a general tweet.

Sparse nodes are essentially nodes with only a connection to the original tweet, without any other possible connection derivable from temporal and social relationships. Since the objective of the proposed technique is estimating possible chains of retweets starting from the partial information provided by the Twitter API, decreasing the incidence of the sparse node means increasing the amount of available information.

#### Definition 21

*(Retweet source incidence)* Given a PCN $\mathcal {N} = \langle \mathcal {X}, \mathcal {C} \rangle$, the *Retweet Source Incidence* (*RSI*) is the number of constraints in which the degree of ownership associated to a retweet source (i.e., derived connection) is greater than the degree of ownership associated to the original tweet, divided by the overall number of retweets. Given the set *RT* defined as:

$$\begin{aligned}&RT = \{\langle T_i, T_j, C_{ij} \rangle \in \mathcal {C} \mid T_{i}. \textsf{type} = \textsf{RT} \wedge T_j. \textsf{type} = \textsf{T} \;\wedge \\&\quad \exists (x_h,[s_h,e_h]) \in C_{ij} (x_h = T_{j}. \textsf{user} \;\wedge \\&\quad \exists (x_k,[s_k,e_k] ) \in C_{ij} (x_k \ne T. \textsf{user} \;\wedge \\&\quad s_k > s_h)) \} \end{aligned}$$

which contains the set of *R-T* edges such that the minimum degree of ownership associated to a user different from the original tweet author *u*, is greater than the degree of ownership associated to *u*. The metric *RSI* can be computed as

24 $$\begin{aligned} RSI(\langle \mathcal {X}, \mathcal {C} \rangle ) = \dfrac{|RT|}{|\{x \in \mathcal {X} \mid x. \textsf{type} = \textsf{RT}\}|} \end{aligned}$$

This last metric is used to identify the number of cases in which the proposed technique is able to identify an alternative source for a retweet which is more likely than the original tweet.

### Result evaluation

Given the datasets described in Table 3 and the PCNs illustrated in Table 4, the proposed constraint propagation technique has been applied and the evaluation metrics introduced in Sect. 5.1 have been computed on the resulting networks. The source code has been made publicly available.^2^ and the tests have been performed on a Hadoop Cluster composed of 10 slaves nodes and 1 master node. The results are reported in Table 6 where each column reports the value of the corresponding metric.

**Table 6:** Evaluation metrics computed on the obtained PCNs

Dataset	ACS	SPI	RSI
$D_1$	1.67	0.47	0.74
$D_2$	1.72	0.38	0.54
$D_3$	1.72	0.32	0.59
$D_4$	1.87	0.30	0.70
$D_5$	1.92	0.29	0.69

The results in Table 6 show that even if in the obtained networks some sparse nodes remain (i.e., SPI metric), namely some retweets continue to be connected only to the original tweet, the average constraint size becomes greater than one (i.e., ACS metric). In other words, each non-sparse retweet has been associated to at least another possible source, beside to the original tweet, and the number of cases in which such additional sources have a minimum degree of ownership greater than the original one exceeds the 50%, reaching in some cases the 74% (i.e., RSI metric).

**Table 7:** Execution statistics

Dataset	Tot. time	Map time (s)			Iterations		
	(s)	Min	Max	Avg	Min	Max	Avg
$D_1$	40	11	15	12	2	6	3.16
$D_2$	60	11	18	12	2	6	3.23
$D_3$	75	11	18	12	2	6	3.18
$D_4$	144	12	20	14	2	9	3.13
$D_5$	251	12	26	16	2	9	3.78

Finally, the results in Table 7 report some information about the performances of the proposed algorithm: the total time taken by the job to complete, some statistics about the execution time of the map tasks, and some statistics about the number of iterations. As regards to the amount of time taken by map tasks, we can observe that even if the splits are not completely balanced, the difference between their minimum and maximum execution time is reasonable and very near to the average. The columns labeled as **Iterations** report the number of iterations performed by the Path Consistency Algorithm to achieve convergence. This number is small and on average three or four iterations are sufficient to complete the constraint propagation.

### Comparison and possible applications

As already discussed, the main difference between the proposed solution and the state of the art resides on the fact that the former tries to identify all the possible connections and assigns them a different likelihood measure based on temporal and social relations. Conversely, the latter ones search for only the most likely source, eventually missing possible important connections.

Despite this difference, we can observe that given the PCN resulting from the execution of Alg. 3, it is always possible to retrieve a single chain of retweet by considering for each retweet only the connection induced by the authorship statement with the greatest minimum degree of ownership. More specifically, as highlighted in Sect. 4.3, after the constraint propagation, the edge between a retweet *R* and its original tweet *T* is characterized by an authorship constraint containing an authorship statement for each possible source with the corresponding degree of ownership. Starting from these *R-T* edges and considering only the authorship statements with the greatest minimum degree of ownership, it is possible to identify the most likely connection and build a simple chain of retweet as the one proposed in  [8]. In other words, the proposed solution is not only more informative than the existing ones, since it provides a more complete description about the situation, but it is also flexible enough to be reduced to a classical graph.

#### Example 10

**Fig. 3:**
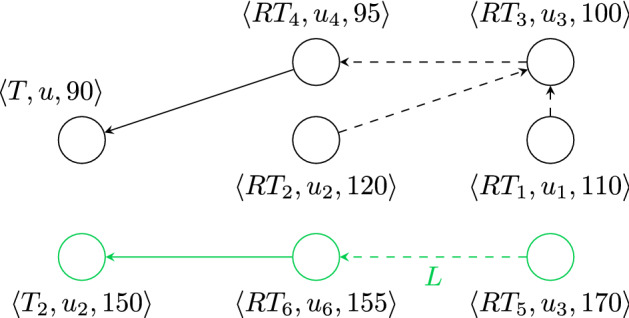
Single chain of retweets obtained from the derived authorship constraints of *R-T* edges

Let us consider the authorship constraints in Table 8 which have been obtained after the application of the constraint propagation to the PCN in Fig. 2. By considering the obtained *R-T* edges and by identifying inside them the most likely authorship statement, we can identify the chain of retweet highlighted in Fig. 3. We take as more likely authorship statement the one with the greatest lower bound in the degree of ownership.

**Table 8:** *R-T* edges for the PCN in Fig. 2 after the application of the constraint propagation algorithm

Edge	Constraint
*A*	$\{(u,[0.16,0.24],(u_1,[0.20,0.29]),(u_3,[\mathbf {0.28},0.47])\}$
*B*	$\{(u,[0.17,0.50]),(u_3,[\mathbf {0.34},0.5])\}$
*E*	$\{(u,[0.33,0.50]);(u_4,[\mathbf {0.34},0.5])\}$
*H*	$\{(u,[\mathbf {0.66},1.00])\}$
*I*	$\{(u_2,[0.22,0.5])\},(u_6,[\mathbf {0.28},0.50])\}$
*M*	$\{(u_2,[\mathbf {0.86},1.0])]\}$

The provided solution can be a more suitable starting point for subsequent analysis. Let us consider for instance the identification of the so called influencer for a given topic, namely the set of users whose contents are the most followed with reference to a given argument. Similarly, given a certain user *u* who retweeted many posts regarding a certain arguments, we can identify that even if the original tweets came from several different sources, *u* actually may take them from only one friend. If we consider only the most likely connection, these analysis can be compromised, because in some cases the identification of the influencers can pass through connections which are not the most likely, as illustrated in the following example.

#### Example 11

**Fig. 4:**
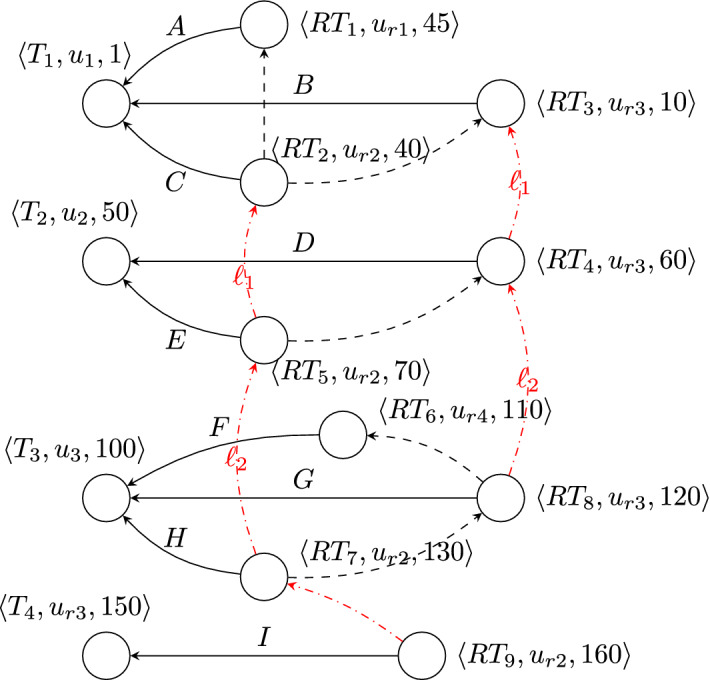
Example of PCN for analyzing $u_2$ behavior

Let us consider the portion of PCN depicted in Fig. 4 where we have 4 tweets regarding the same topic and the set of their retweets. For not cluttering the notation we have labeled only the *R-T* edges, since after the constraint propagation we will consider only their constraints in order to reconstruct the possible retweet sources.

Suppose that we are interested in discovering the behavior of user $u_{r2}$. At first glance, we could observe that $u_{r2}$ has performed several retweet activities by considering various sources of information, and in particular user $u_1$, $u_2$, $u_3$ and $u_{r3}$. Conversely, if we apply the constraint propagation algorithm, we obtain the following authorship constraints for the $u_{r2}$’s retweets:

$$\begin{aligned}&A = \{(u_1,[0.11,0.33]), \mathbf {(u_{r1}, [0.34,0.34])}, (u_{r3},[0.25,0.30]) \}\\&E = \{\mathbf {(u_2,[0.35,0.50])}, (u_{r3},[0.33,0.50]) \} \\&H = \{(u_3,[0.17,0.5]),\mathbf {(u_{r3},[0.46,0.50])} \} \\&I = \{(u_{r3},[0.0,1.0]) \} \end{aligned}$$

and we can observe that there is a user in common in all the authorship constraints, namely $u_{r3}$. Therefore, it is more likely that $u_{r2}$ actually takes as his/her source of information $u_{r3}$, instead of several sources. This connection could be missed in case we apply a traditional reconstruction approach, since $u_{r3}$ is not associated to the authorship statement with the greatest degree of ownership (due to the relative temporal distance the most likely connection is the $RT_3$), so this connection will be likely discarded.

## Conclusion

As social media becomes more and more important as source of information, the need for tracking and providing a complete description of social provenance of news becomes a crucial activity. The Twitter platform is considered one of the most important social sources of news, thanks also to the presence of mechanism able to easily and rapidly share them, such as retweets, quotes and reply posts. However, the Twitter API does not provide complete information about chains of retweets yet, since it only stores the connection between a retweet and its original post. This can significantly reduce the ability to provide a complete inference of the social provenance and also further analysis about user interactions and the mutual influence among users. For all these reasons, this paper provides an innovative solution for generating a complete retweet cascade graph, which differs from the other available approaches since it tries to reconstruct all possible connections between tweets and assigns them a weight proportional to their temporal and social relationship. This approach is based on the notion of Provenance Constraint Network (PCN), a data structure inspired by the well-known Temporal Constraint Network, and an adapted version of the Path Consistency Algorithm for authorship constraint propagation. A MapReduce implementation of the proposed technique is provided and an experimentation on a real-world dataset is presented. The obtained results highlight the potentiality of the proposed approach in particular as regards to the possibility to apply more refined analysis. As future work we plan to investigate our approach combined with sentiment analysis techniques, in order to consider their potentialities in the analysis of textual contents of tweets and quotes and for refining the concept of social interaction between users.

## Data Availability

For the experimental phase, we consider a portion of dataset provided in [26] which contains a large collection of COVID-19-related tweets collected starting from January 2020.

## Footnotes

1 https://help.twitter.com/en/using-twitter/types-of-tweets.

2 https://github.com/smigliorini/pcn.

## Code Availability

The source code is available at https://github.com/smigliorini/pcn.
